# Toward a systematic understanding of cancers: a survey of the pan-cancer study

**DOI:** 10.3389/fgene.2014.00194

**Published:** 2014-07-03

**Authors:** Zhaoqi Liu, Shihua Zhang

**Affiliations:** National Center for Mathematics and Interdisciplinary Sciences, Academy of Mathematics and Systems Science, Chinese Academy of SciencesBeijing, China

**Keywords:** cancer genomics, pan-cancer study, data integration, The Cancer Genome Atlas, bioinformatics

## Abstract

Studies on molecular aberrations of cancer patients have increased unprecedentedly in scale and accessibility, allowing large-scale integrative cross-cancer analysis. Pan-cancer study is becoming a valuable paradigm for cancer genomics. Here, we review recent advances in this field and highlight the potential challenges and directions especially from the computational angle.

## Introduction

Cancers have been believed as complex genomic diseases nowadays. They are largely caused by molecular aberrations including somatic mutations, copy number alterations, transcriptional expression changes, epigenetic variations, and so on. Great advances in high-throughput techniques and comprehensive efforts have revealed a systematic investigation of the genomic landscapes of human cancer.

The Cancer Genome Atlas (TCGA) project started in 2005 with the goal of profiling and analyzing more than 10,000 tumor samples from about 20 tumor types, provides an unprecedented opportunity to systematically analyze molecular aberrations of cancer through the application of genomic technologies. For each individual cancer type, the rich molecular data from six types of omics platforms were analyzed and integrated to identify novel oncogenic drivers, establish molecular subtypes and discover new biomarkers (McLendon et al., 2008; Bell et al., 2011; Muzny et al., 2012; Hammerman et al., 2012; Koboldt et al., 2012; Creighton et al., 2013; Kandoth et al., 2013b). These comprehensive analyses have identified many important genomic similarities among tumor types and subtypes, which present an opportunity to design tumor treatment strategies and enable therapeutic discoveries among tumors regardless of tissue or organ of origin. This suggests the potential importance of developing a comprehensive analysis across cancers to find the pan-cancer similarities and tumor-specific characteristics.

**Figure 1:**
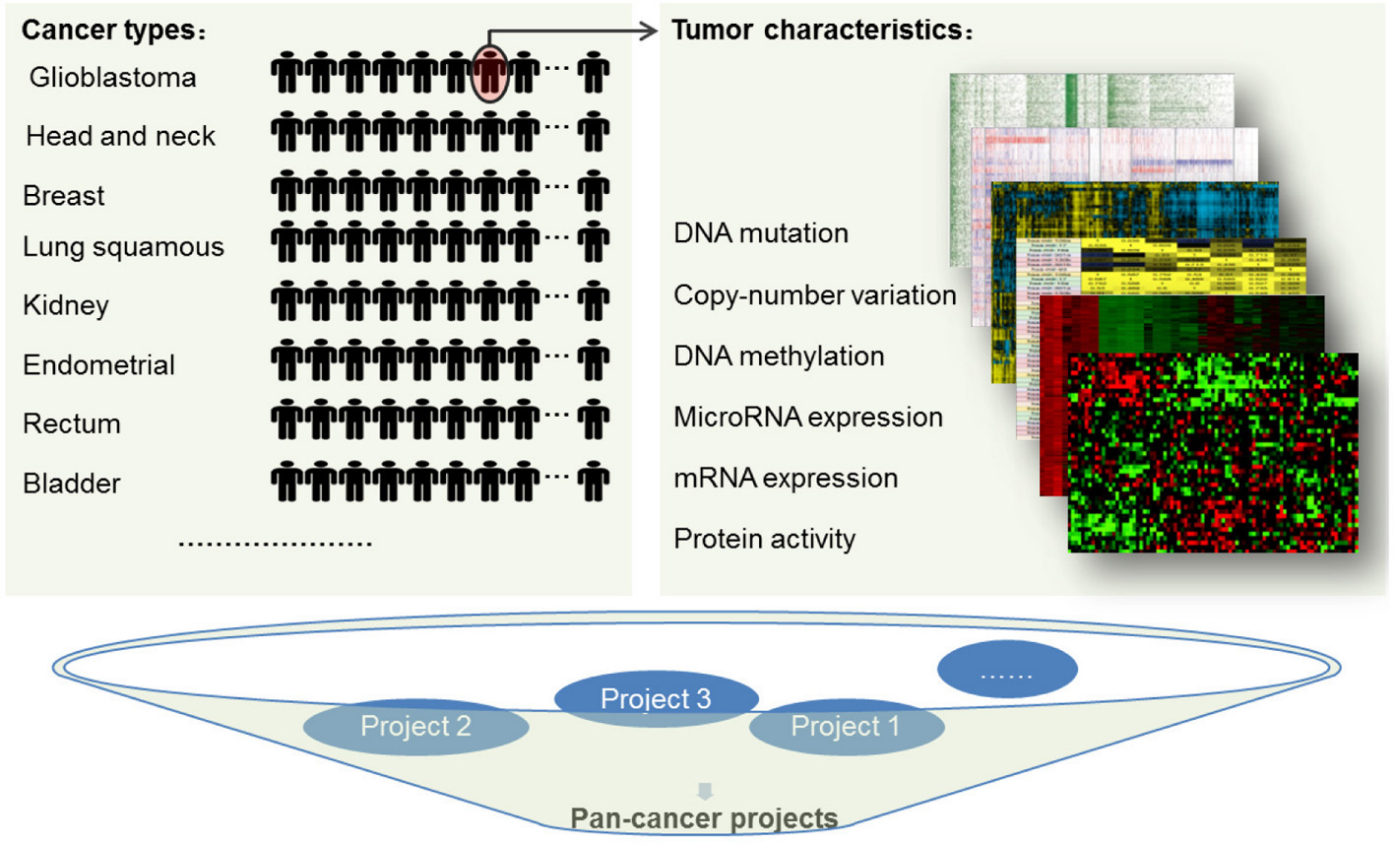
**Multiple omics datasets from diverse tumor types enable comprehensive analyses on numerous aspects of cancer genomics**. The pan-cancer data includes rich biomolecular profiles on six types of platforms (mutation, copy number, methylation, gene expression, microRNA and reverse phase protein arrays) from tumors occurring in different sites of the body (glioblastoma, lymphoblastic acute myeloid leukemia, head, and neck squamous carcinoma, lung adenocarcinoma, lung squamous carcinoma, breast cancer, kidney renal, clear-cell carcinoma, ovarian carcinoma, bladder carcinoma, colon adenocarcinoma, uterine cervical and endometrial carcinoma, and rectal adenocarcinoma).

To this end, TCGA launched the Pan-Cancer analysis project to compare the molecular data of 12 tumor types (Figure 1). The pan-cancer study aims to examine the similarities and differences among the genomic and cellular alterations across diverse tumor types by analyzing multiple profiles of large number of human tumors. The first batch of research achievements of this promising project have been released very recently (Ciriello et al., 2013; Kandoth et al., 2013a; Lawrence et al., 2013; Weinstein et al., 2013; Zack et al., 2013). The recently published studies based on these comprehensive datasets provide more systematic understanding of human cancer on genomic, epigenomic, transcriptomic, proteomic, and clinical levels. The majority of these findings focus on cancer genomic variations such as somatic mutation, copy number alteration, and chromosomal aberrations. Although the pan-cancer project has made great advances, deciphering the complicated data in meaningful terms is still in its early stage. In this paper, we mainly review the progresses of Pan-cancer project, discuss high-related “pan-cancer” studies and highlight potential challenges and directions (Table 1).

**Table 1:** **Brief summary of recent pan-cancer studies**.

	**p/t**	**Resources**	**dt**	**Summary**	**References**
1	3281/12	TCGA	S	Describe variable mutation frequencies and contexts and their links to environmental factors and defects in DNA repair, and identify 127 significantly mutated genes.	Kandoth et al., 2013a
2	7042/30	TCGA, ICGC, and others	S	Extract 21 distinct mutational signatures, find some present in many cancers and certain ones are associated with phenotypic features, and discover localized hypermutation “kataegis” in many cancers.	Alexandrov et al., 2013
3	3185/12	TCGA	S	Analyze known phosphorylation sites mutated by single nucleotide variants, predict signaling-specific cancer driver genes, and create a high-confidence collection of cellular signaling-related cancer mutations.	Reimand et al., 2013
4	4632/13	TCGA, ICGC, and others	S	Propose a platform for summarizing somatic mutations, genes and pathways involved in tumorigenesis, and identifying, ranking, and visualizing cancer drivers.	Gonzalez-Perez et al., 2013b
5	5277/19	TCGA	SE	APOBEC3B is the most likely cause of a large fraction of both dispersed and clustered cytosine mutations in six distinct cancers.	Burns et al., 2013
6	3205/12	TCGA	S(C)	Employ five complementary methods to search for mutational driver genes, demonstrate its advantage, and provide a list of 291 high-confidence cancer driver genes.	Tamborero et al., 2013
7	3083/27	TCGA	S	Demonstrate the false-positive cancer gene identification issue, provide a methodology MutSigCV to eliminate the artifactual findings and enable the identification of true cancer associated genes.	Lawrence et al., 2013
8	2680/14	TCGA, dbGaP, and others	S	Demonstrate a significant presence of the APOBEC mutation pattern in certain cancers.	Roberts et al., 2013
9	4742/21	TCGA, dbGaP	S	Find that large-scale genomic analysis can identify nearly all known cancer genes, report 33 novel genes, conduct down-sampling analysis and estimate the tumor number of samples for near-saturation.	Lawrence et al., 2014
10	4934/11	TCGA	C	Compare patterns of copy number change across cancer types, determine individual SCNA events and their temporal ordering from these profiles and identify functionally relevant correlations between SCNAs.	Zack et al., 2013
11	8227/19	GEO	C	Discover similarity of chromosomal arm-level alterations and co-occurring pairs of arm-level alterations, identify cancer-related gene enriched recurrent focal alterations, and tumor type-specific alterations with enriched functional categories.	Kim et al., 2013
12	3290/11	TCGA	RE(CM)	Infer recurrent cancer-associated miRNA-target relationships across multiple cancer types, which were highly consistent with published data from miRNA perturbation experiments and predictions based on sequencing technology.	Jacobsen et al., 2013
13	4186/11	TCGA, AGO-CLIP	MCRE	Describe a pan-cancer co-regulated oncogenic microRNA “superfamily,” define mutations in microRNA target sites, and identify pan-cancer oncogenic co-targeting pathways by the miR-17-19-130 superfamily members.	Hamilton et al., 2013
14	82 cell lines	ENCODE	ME	Provide an atlas of DNA methylation across diverse samples, enable new discoveries about DNA methylation and its role in gene regulation and disease.	Varley et al., 2013
15	4379/11	TCGA	P	Develop a user-friendly data portal, The Cancer Proteome Atlas (TCPA) with six modules: Summary, My Protein, Download, Visualization, Analysis, and Cell Line.	Li et al., 2013
16	2920/11	TCGA and other 31 datasets	E(CS)	Describe a method called “ESTIMATE” that uses gene expression signatures to infer the fraction of stromal and immune cells in tumor samples.	Yoshihara et al., 2013
17	4433/19	TCGA	E	Screen for expressed viruses across diverse cancers, provide a large-scale virus–tumor association map, and confirm and extend current knowledge.	Tang et al., 2013
18	3299/12	TCGA	SCM(E)	Develop an algorithmic approach to hierarchically stratify tumors, divide tumors into two major classes, and reveal oncogenic signatures to characterize tissue-independent subclasses of tumors.	Ciriello et al., 2013

*p/t, number of patients/number of tumor types; Resources, major data resources; dt, major molecular data types used for pan-cancer study and validation analysis in bracket including somatic mutation (S), copy number variation (C), DNA methylation (M), microRNA expression (R), mRNA expression (E) and reverse-phase protein arrays (P); Summary, summary of the key contributions*.

## Somatic mutations

Somatic mutations are essential for tumorigenesis and most human cancers are caused by a small number of driver gene mutations that develop over the course of about two decades (Vogelstein et al., 2013). Therefore, a comprehensive investigation of the mutational landscape of multiple cancer types would definitely be a critical basis for cancer diagnostics, therapeutics, and selection of rational combination of therapies. Finding the driver mutations from passengers will still be the major challenge in cancer genomics. Large-scale genomic analysis and approaches that use cross-tumor principles definitely enable the identification of validated and novel driver genes while dramatically improving the sensitivity and efficiency compared to the traditional calls by individual-tumor-type research (Lawrence et al., 2013).

Kandoth et al. reported 127 significantly mutated genes from diverse cellular processes across different tumor types, and identified cancer type-specific signatures of driver mutations in several dominant cancer types (Kandoth et al., 2013a). They found that kidney renal clear cell carcinoma (KIRC) has the strongest exclusivity from the other 11 cancer types with high mutation frequency of *VHL* and *PBRM1*. However, besides *TP53*, there was hardly any common mutation shared by multiple cancer types based on the observation of the reported genes, disenabling the discovery of potential extending of shared effective treatments among tumors. They pointed that the combination of drivers varies for individual patients in each cancer type and it was crucial for optimizing the treatment. Lawrence et al. conducted a large-scale genomic analysis of somatic point mutations in exome sequences from 4742 human cancers and identified nearly all known cancer driver genes and 33 novel candidates (Lawrence et al., 2014). However, more validations on these new drivers are required with experimentally follow-up studies.

Phosphorylation has been considered as an important factor in cancer which is involved in key processes such as the control of proliferation, oncogenic kinase signaling. It was recently reported that cancer may be driven by statistically significant and spatially specific mutations in protein sites involved in cellular phosphorylation signaling (Reimand and Bader, 2013). More recently, Reimand et al. extended their study to detect such mutations to 3185 tumor genomes across 12 cancer types, and predicted 54 additional cancer-specific drivers and 82 genes only seen in pan-cancer analysis (Reimand et al., 2013). However, this analysis only restricted known signaling alterations to protein-coding mutations which only comprise a minority of all cancer mutations, limiting the extent of mutated signaling in tumor cells caused by other mechanisms.

It has been demonstrated that computational analyses of sequence data for identifying driver mutations from large cohorts of tumor samples are not trivial due to the heterogeneous nature of cancer and all existing methods for the identification of genes exhibiting signals of positive selection show particular shortcomings and specific biases (Gonzalez-Perez et al., 2013a). Recently, Tamborero et al. proposed an integrative strategy to combine five complementary methods which enables the identification of a comprehensive and high-confident pan-cancer driver gene list (Tamborero et al., 2013). This analysis have shown that the combination of complementary methods are effective than individual methods. However, there is no gold-standard dataset of driver and passenger genes to assess the quality of such combination. Thus, it naturally introduces a computational issue that what the reasonable or optimal combination of different methods is. Practical exploration on the composition and structure of the investigated genomic dataset and detailed learning on the principle of each method would help to form a better combination analysis than traditional intuitive operation, e.g., combining the output *p*-values, or overlapping the top-ranking genes from diverse algorithms.

The investigation of temporal relationship of somatic genetic events would provide new insights into the discovery of driver oncogenes. It is reported that the timing of vital mutation is likely to be related to metastasis, which is responsible for the death of most patients with cancer. The genetic changes that occur early during malignant transformation may represent promising targets for therapeutic intervention (Vogelstein et al., 2013). Thus, a comprehensive analysis of determining the temporal sequence of somatic genetic events would help the identification of important mutations across 12 cancer types, which was untouched extensively by previous studies. This is probably because the lack of effective computational algorithms (Attolini et al., 2010). More efforts and techniques are needed in developing fast and accurate models to resolve this issue. Moreover, the identification of genetic alterations that leads to cancer metastasis is remarkably limited still now and need to be further studied with the abundant pan-cancer data.

In order to reveal the causes of extensive somatic mutations accrued in cancers, a global analysis with the pan-cancer dataset found that APOBEC3B-catalyzed genomic uracil lesions are responsible for a large proportion of mutations in distinct cancer types (Burns et al., 2013). Cytidine deaminases, which convert cytosine bases to uracil during RNA editing, may contribute to DNA damage. A similar study showed a significant presence of the APOBEC mutation pattern in bladder, cervical, breast, head and neck, and lung cancers (Roberts et al., 2013). Meanwhile, a newly introduced concept of understanding the biological processes generating mutations, mutational processes, were explored on the TCGA, ICGC and other datasets using a previously developed computational framework. Finally, they extracted more than 20 distinct mutational signatures, one of which attributed to the former mentioned APOBEC family of cytidine deaminases (Alexandrov et al., 2013). In addition, hypermutation localized to small genomic regions called “kataegis” was found in many cancer types.

All these comprehensive analyses on the mutation profiles have proven the enhanced ability of detecting driver genes with the increase in the number of patients across 12 tumor types. However, cancer is a disease of pathways driven by underlying systematic alterations. The main subjects of alterations are not individual driver genes, but rather modules of functionally related proteins at pathway-level. With an increase in the number of mutational profiles across different tissues, critical and tumorigenesis-associated pathways would be discovered to enable physicians to select the best combination therapy for each patient. To provide an exhaustive description of potentially actionable pathway-level catalog of the driver mutations would be a challenge for specific targeted therapeutics across cancer types.

**Figure 2:**
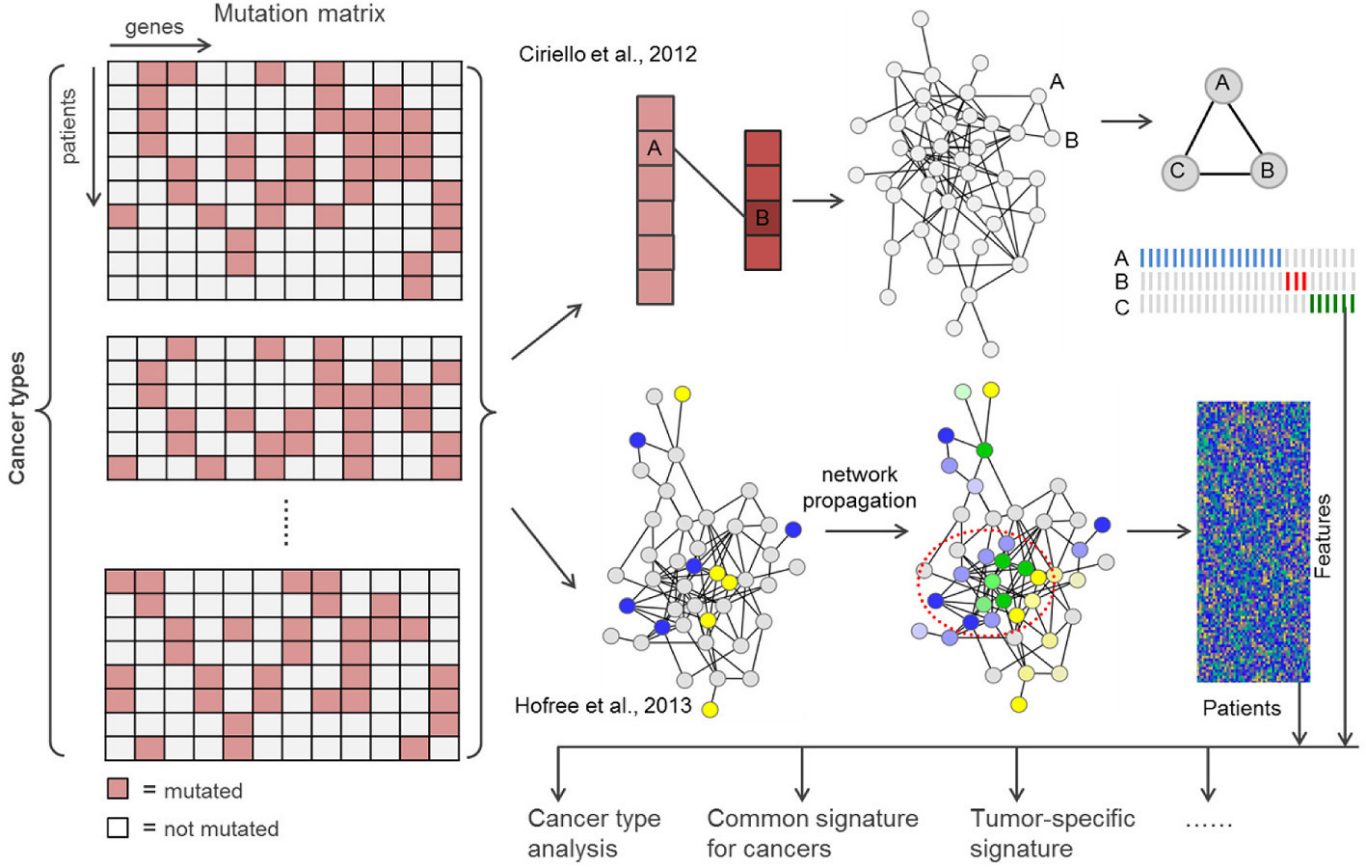
**Two potential network-based frameworks for cross-tumor comparative analysis and tumor-specific feature discovery**.

Computational methods for integrating, comparing and interpreting genome-scale molecular information are urgently needed in current stage and known algorithmic approaches may be adopted and adapted for such analysis. For example, to identify mutated driver pathways using somatic mutation data, Vandin et al. developed a method by considering mutual exclusive principle and high coverage (Vandin et al., 2012). Zhao et al. proposed a powerful mathematical programming model to solve it and suggested to incorporate gene expression data to prioritize the true functional ones (Zhao et al., 2012). Ciriello et al. proposed a network-based method to detect driver modules that obey the mutual exclusivity principle (Ciriello et al., 2012). Hofree et al. devised a network-based approach to integrate discrete somatic mutation data with known biological molecular networks to stratify patients into subtypes for individual tumor types (Hofree et al., 2013). We believe that the similar network-based framework can be adopted for cross-tumor comparative analysis and finding tumor-specific features (Figure 2).

## Somatic copy number alterations

Somatic copy number alterations (SCNA) are ubiquitous and affect a majority of the genome in cancers. It has been comprehensively demonstrated that SCNAs play critical roles in activating oncogenes and in inactivating tumor suppressors. Distinguishing the driver events from the passenger SCNAs, identifying their gene targets and describing their functional roles are major challenges in current stage. The unprecedented large-scale data of copy number profiles across cancers will enable the identification of recurrent chromosomal alterations with potential clinical benefits, provide more systematic understanding of human cancer, and leads to substantial advances in cancer diagnostics and therapeutics.

In a recent study, Zack et al. conducted a comprehensive analysis of high-resolution copy number profiles of the TCGA data and identified common patterns of SCNA across cancer types (Zack et al., 2013). They found that whole-genome doubling observed in 37% of cancers was associated with higher rates of every other type of SCNA. They suggested that the diverse lengths of SCNAs in the middle of chromosomes and those of the telomere-bounded ones indicate different mechanisms of generation. They reported a number of significantly recurrent focal SCNAs in 140 regions, some of which were enriched for genes involved in epigenetic regulation, or encompassed genes tending to generating interacting protein product. In another study, Kim et al. found that chromosomal arm-level alterations among developmentally related tumor types tend to be similar (Kim et al., 2013). They also reported a number of co-occurring pairs of arm-level alterations, and found that recurrent pan-cancer focal alterations are enriched with known cancer related genes. Both of these two studies explored the rare cataclysmic event that occurs in a small fraction of chromosomes called chromothripsis. Specifically, a number of localized chromothripsis events associated with known cancer-related genes were revealed on chromosome 2 in some neuroblastoma cases (Kim et al., 2013). Similarly, Zack et al. revealed that chromothripsis was detected in 16% of glioblastomas, reaching the highest rate across cancers (Zack et al., 2013). The fact that arm-level alterations tend to be shared among diverse cancer types was also reported in a previous study (Beroukhim et al., 2010), where 158 regions of focal somatic copy-number alterations were identified and a large majority of them were present at significant frequency in multiple cancer types. Such large-scale analysis provides insights into mechanisms of generation and functional consequences of cancer-related SCNAs, which cannot be revealed directly in individual cancer.

However, due to the intrinsic complexity of cancer genomes, powerful algorithmic approaches are still needed for deep exploration of the large-scale copy number alteration profiles for driver events and the chromothripsis based on the integration of new data resources. Statistical analysis of co-occurrence and mutual exclusivity of genomic aberrations were needed to be further explored. Akavia et al. once developed a computational framework to integrate chromosomal copy number and gene expression data for identifying cancer drivers based on the hypothesis that copy number aberrations often influence the expression of genes in a module via changes in expression of the driver (Akavia et al., 2010). Extending of such computational framework for identifying robust common drivers and cancer-specific ones will be promising in the near future.

Recently, a novel algorithmic approach that uses 479 selected functional events obtained from significance analysis on the mutation, copy number variation and DNA methylation profiles derived a hierarchical classification of 3299 TCGA tumors from 12 cancer types (Ciriello et al., 2013). The top two classes of the clusters are dominated by mutations (M class) and copy number changes (C class) respectively, which would be due to the treatment of the mutation and copy number features equally and separately. The M class of tumors contained almost all the samples in kidney clear-cell carcinoma, while almost all ovarian cancer fell into the C class. Patients within the same subclass may be benefited from the observed cross-cancer distribution of targetable events. Integrating these three kinds of significant features with different weights on gene-levels would provide diverse findings and the ready-processed data matrix of selected functional events will be valuable for other related analysis.

## DNA methylation alterations

DNA methylation is a key determinant of regulatory chromatin complexes that has a complex relationship with gene expression and was found to be dysregulated in many cancers. Recently, a large-scale DNA methylation study on 82 human cell lines and tissues provides an atlas of DNA methylation across diverse and well-characterized samples and enables new discoveries about DNA methylation and its role in gene regulation and disease (Varley et al., 2013). The comparisons of methylation profiles across different cancer cell lines identified cancer-associated and cell-type specific methylation signatures. The relationship between DNA methylation and gene expression levels were well observed across the genome; however, evidences of its directed or indirected associations with other molecular and phenotypic characteristics across multiple cancers are of potential interest and are worth further exploring with the aid of pan-cancer data.

## MicroRNA and gene expressions

MicroRNAs (miRNA) have been demonstrated to play key roles in gene regulation by binding target mRNAs in a sequence complementary manner. Previous studies have shown that dysregulation of microRNAs can contribute to tumor formation and progression. Recently, Jacobsen et al. explored the common processes of tumor biology regulated by microRNAs across 11 diverse cancer types (Jacobsen et al., 2013). They adopted a multivariate linear regression model to evaluate a causal relationship score of each pair of miRNA and mRNA in individual cancer types and employed a rank-based statistical method to integrate scores obtained from multiple cancer types to infer recurrent pan cancer-associated miRNA-mRNA relationships from miRNA and mRNA expression profiles. The predicted miRNA-target interactions were shown to be highly consistent with published experimental data and computational predictions, and form a high-confidence pan-cancer network of 143 recurrent target relationships for further analysis. Computationally, this current analysis didn't address the potential nonlinear effect between miRNA and mRNA which need to be addressed further.

In another study, Hamilton et al. explored the microRNA regulatory landscape and identified pan-cancer microRNA drivers of cancer by integrating the TCGA Pan-Cancer microRNA, mRNA, copy number variation (CNV) and exome-sequencing data sets from 12 tumor types with a miRNA target atlas composed of publicly available Argonaute Crosslinking Immunoprecipitation (AGO-CLIP) data (Hamilton et al., 2013). They showed a pan-cancer, coregulated oncogenic microRNA “superfamily,” which cotargets critical tumor suppressors via a central GUGC core motif. Through these two integrative pan-cancer analysis frameworks, we were able to understand microRNA regulatory architectures across multiple tumor types. Both studies have shown new examples of miRNAs that coordinately regulate cancer pathways across many cancer types, demonstrating the potential roles of miRNA-target co-modules. In the future studies, computational techniques for identifying such co-modules (Zhang et al., 2011) can be developed to pan-cancer data set for exploring common ones across diverse cancer types directly.

In addition, other pan-cancer related works include a method that uses gene expression signatures to infer the fraction of stromal and immune cells in tumor samples (Yoshihara et al., 2013), and a landscape of virus–tumor map generated using transcriptome sequencing data (Tang et al., 2013). Actually, 10 year ago, Segal et al. have conducted a study to address the commonalities and variations between different types of tumor using DNA microarrays (Segal et al., 2004). They implemented an integrated analysis of 1975 published microarrays spanning 22 tumor types. They defined co-expression modules based on expression profiles in different tumors and employed a unified analysis to characterize gene-expression profiles in tumors with activated and deactivated modules. They have found that activation of some modules is specific to particular types of tumor and other modules are shared across a diverse set of tumors. We believe that the revisit of the large-scale pan-cancer study in terms of expression profiles and integration analysis with other genomics data will improve the understanding for diagnostic, prognostic and therapeutic studies.

## Web tools for pan-cancer study

Several useful web tools have been developed to interactively visualize and explore the large-scale TCGA pan-cancer data (Table 2). Specifically, Gonzalez-Perez et al. developed a web platform called IntOGen-mutations to identify and visualize cancer drivers across tumor types, which provides convenience for better clinical decision-making (Gonzalez-Perez et al., 2013b). Moreover, Li et al. developed a user-friendly data portal with six modules, The Cancer Proteome Atlas (TCPA), which provides comprehensive, and unique cancer proteomic data and powerful visualizing and analysis modules for exploring such data (Li et al., 2013). Jacobsen et al. have presented all predictions of miRNA-target relationships in their study on an online resource, which allows exploration, prioritization and visualization of novel miRNA-target interactions in TCGA data (Jacobsen et al., 2013). The University of California Santa Cruz (UCSC) Genome Browser has become a very important tool which offers online public access to a growing database of genomic sequence and annotations for a large collection of organisms and provides an integrated environment for visualizing, comparing, analyzing and sharing both publicly available and user-generated genomic data sets with various web-based tools. Cline et al. has extended this powerful Browser to explore the impact of genomic alterations on phenotypes by visualizing data of different platforms and levels, performing cancer classifications and conducting patient survival analysis (Cline et al., 2013). The Synapse web server developed by Sage Bionetworks is an informatics platform of public resources for the scientific community and encourages scientists to discover and share data, models, and analysis methods. The TCGA pan-cancer group has collaborated on this system to share and evolve data, results, and methodologies throughout the full duration of the project (Omberg et al., 2013). More importantly, updates of new datasets and discoveries will be immediately available based on this system. In summary, all these resources and tools will provide great convenience and promote pan-cancer type of study.

**Table 2:** **Brief summary of useful webserver or database for pan-cancer study**.

**Name**	**Website**	**Key purposes**	**References**
IntOGen-mutations	http://www.intogen.org/mutations	Identify and visualize cancer drivers across tumor types.	Gonzalez-Perez et al., 2013b
CancerMiner	http://cancerminer.org	Search recurring microRNA-mRNA associations across cancer types.	Jacobsen et al., 2013
Synapse	https://www.synapse.org/	Collaborate with the TCGA pan-cancer group to share and update data, results and methodologies.	Omberg et al., 2013
TCGA	http://cancergenome.nih.gov/	Provide a platform for researchers to search, download, and analyze data sets generated by TCGA.	Weinstein et al., 2013
TCPA	http://bioinformatics.mdanderson.org/main/TCPA:Overview	Facilitate access of the broader research community to cancer proteomics datasets.	Li et al., 2013
UCSC Cancer Genomics Browser	https://genome-cancer.ucsc.edu	Offer interactive visualization and exploration of TCGA genomic, phenotypic, and clinical data.	Cline et al., 2013

## Discussion and conclusion

Although several previous pan-cancer studies focusing on multiple tumor types or cell lines have been reported before (Segal et al., 2004; Lee et al., 2008; Sahin et al., 2008; Wu et al., 2010; Beroukhim et al., 2010), the ongoing pan-cancer project has provided an unequaled resource for the integrative analysis of multiple cancer types, and achieved remarkable discoveries. Generally, the main investigation and observations are attributable to two fundamental aspects: intra-cancer heterogeneity and cross-cancer similarity reflected in different levels of molecular properties. However, along with these progresses, new challenges are emerging and pressing to be resolved.

How to integrate the data generated on different platforms or different versions of the same platform is an unavoidable challenge which doesn't account for the challenge in the integration of data across cancer-types. Consensus and reliable standardization of the input data will be a key step to obtain robust and reliable results from the true biological signals and conquer the unwanted batch effects. Large-scale collaborative analysis and open community-based competition has been suggested to be one possible solution to establish best practices for overcoming this challenge (Omberg et al., 2013).

To our knowledge, there were no well-established and unified approaches to integrate different molecule data in pan-cancer studies. There were only some general routine techniques such as robust quantile normalization or *z*-score transformation for conquering the data scale issue of different cancer datasets. Currently, most published pan-cancer studies prefer to rerun the same algorithm on each cancer type individually and compare or combine the results to derive the pan-cancer similarities in statistical fashion or meta-analysis.

The multi-dimensional genomic profiling data provide unique opportunities to study the coordination between regulatory mechanisms on multiple levels. Recently, we have developed methods for the integrative analysis of multi-dimensional genomics data and the discovery of underlying combinatorial patterns (Li et al., 2012; Zhang et al., 2012). The discovered multi-dimensional modules have been demonstrated to reveal perturbed pathways that would have been overlooked with only a single type of data, uncover associations between different layers of cellular activities and allow the identification of clinically distinct patient subgroups. It will be valuable to adopt such study to uncover hidden patterns of multi-dimensional “omics” data across tumor types.

Due to the shared molecular dimension, the pan-cancer studies are focusing on the molecular properties. However, unlike molecular profiles, most clinical features are incomparable across tumor types due to the nature and availability of such data (Weinstein et al., 2013). For example, tumor stage and grade are not comparable as each tumor has its own system. Furthermore, some clinical features are collected according to the classification by tissue or organ, making them vary widely across tumors. Thus, how to effectively employ the clinical features in performing comparative analysis involving multiple cancers will be an important but challenging issue.

Almost all the pan-cancer studies are involved in direct use of existing computing techniques, or previously well-developed approach with an extending analysis on the new dataset. However, the uniqueness and complexity of the pan-cancer data may require more specific and modified approaches for novel discoveries of underlying principles in tumor evolution. Moreover, in contrast to well-studied phenotypic heterogeneity in tumors, the genetic heterogeneity among the cells of an individual tumor or tumors of different patients set an obstacle to effective response to uniformly designed therapeutics. This issue could not be simply resolved by numerical calculation. More efforts on personalized medicine and development of treatment should take advantage of detection of this heterogeneity. For example, Liu et al. evaluated the patient survival prediction performance of genomic and clinical data on the five intrinsic breast cancer subtypes and revealed that molecular gene profiles and clinical features have different prognostic power (Liu et al., 2014). How to extend this kind of studies to make a pan-cancer type of analysis will be an interesting and meaningful problem.

Distinguishing and interpreting the functional role of variants in the noncoding parts of the sequences is an open frontier which has not been as well explored so far. In addition, the prediction of the functional consequences of chromosomal- scale structural variation are also challenging (Weinstein et al., 2013; Wheeler and Wang, 2013).

The ultimate goal of almost all cross cancer studies is to affect clinical decision-making, accelerate the discovery of novel therapeutic agents applied for tumors rising from different organs with similar genomic characteristics. The number of commercially available targeted cancer drugs is still limited nowadays. New computational findings require rapid and effective methods for functional validation. Experimental follow-ups are always critical to assess the hypotheses and consequences. Therefore, a great challenge is how to speed up the process of translating novel discoveries into treatments based on experimental measurements.

As we known that, the process of tumor usually take decades to develop but cancer metastasis occurs only a few years before death. Thus, the investigation on molecular aberrations account for cancer metastasis should be highly informative. Moreover, the knowledge learned from cancer genomics can also be exploited to develop methods for prevention and early detection of cancer, which will be essential to reduce cancer morbidity and mortality (Vogelstein et al., 2013). Besides, the causal relationships of several carcinogenic etiologies with multiple cancer types are also worth exploration (Weinstein et al., 2013). All these challenges enable the pan-cancer study to be a hot topic.

## Author contributions

Shihua Zhang and Zhaoqi Liu conceived this study and wrote the paper.

### Conflict of interest statement

The authors declare that the research was conducted in the absence of any commercial or financial relationships that could be construed as a potential conflict of interest.
